# Reducing contrast media and radiation dose in CT angiography at low tube voltage: animal study with photon-counting detector CT

**DOI:** 10.1186/s41747-025-00577-y

**Published:** 2025-03-24

**Authors:** Konstantin Klambauer, Thomas Flohr, Lukas Jakob Moser, Victor Mergen, Matthias Eberhard, Andreas Prokein, Hatem Alkadhi, Hubertus Pietsch, Gregor Jost

**Affiliations:** 1 Diagnostic and Interventional Radiology, University Hospital Zurich, University of Zurich, Zurich, Switzerland; 2 Department of Radiology and Nuclear Medicine, Maastricht University Medical Center, Maastricht, The Netherlands; 3 Siemens Healthineers AG, Forchheim, Germany; 4 MR and CT Contrast Media Research, Bayer AG, Berlin, Germany

**Keywords:** Computed tomography angiography, Contrast media, Low tube voltage, Photon-counting detector, Radiation dose

## Abstract

**Background:**

Reducing radiation and contrast media (CM) doses in computed tomography angiography (CTA) is especially relevant for potentially vulnerable populations. Low tube voltage photon-counting detector CT (PCD-CT) offers an improved iodine contrast-to-noise ratio (CNR) as compared to conventional CT scanners. We investigated optimized radiation and CM doses of PCD-CT angiography at low tube voltage in an animal model.

**Methods:**

Six minipigs (median weight: 32.5 kg; IQR: 29.8–34.6 kg) underwent thoracoabdominal CTA using a clinical dual-source PCD-CT at 70 kVp with three scan protocols: (A) reference (100% CM and radiation dose), (B) increased radiation (233%) and reduced CM (56%) dose, and (C) reduced radiation (50%) and increased CM (141%) dose. CNR, subjective image quality, and radiation doses were assessed, with statistical analysis including Mann–Whitney *U*-test and Kruskal–Wallis tests.

**Results:**

CTDI_vol_ was 1.7 mGy (IQR: 1.5–1.8) for scan A, 4.3 mGy (IQR: 3.8–4.7) for scan B, and 0.9 mGy (IQR: 0.8–1.0) for scan C (*p* < 0.001). CM volumes were 16 mL (IQR: 15–17) for scan A, 10 mL (IQR: 8–10) for scan B, and 23 mL (IQR: 21–24) for scan C. No significant differences in CNR were found between scans, with medians of 26 (IQR: 24–28) for scan A, 23 (IQR: 22–26) for scan B, and 26 (IQR: 24–30) for scan C (*p* = 0.276). Subjective image quality was similar across scans (*p* = 0.342).

**Conclusion:**

Low tube voltage PCD-CT angiography allows substantial reductions in radiation and CM dose while maintaining stable and improved CNR, which allows further dose flexibility for individualized CTA protocols.

**Relevance statement:**

PCD-CT at low tube voltage provides a high CNR and great flexibility in dose optimization, making it particularly effective for applications where minimizing radiation and CM exposure is a priority.

**Key Points:**

- Low tube voltage imaging with photon counting detector (PCD)-CT enables flexible contrast and radiation dose optimization strategies in thoracoabdominal CT angiography (CTA).
- The CNR for thoracoabdominal CTA remains stable with appropriate contrast and radiation dose adjustments at low tube voltage PCD-CT.
- Low tube voltage PCD-CT consistently yields diagnostic image quality in thoracoabdominal angiography even at reduced contrast or radiation doses.

**Graphical Abstract:**

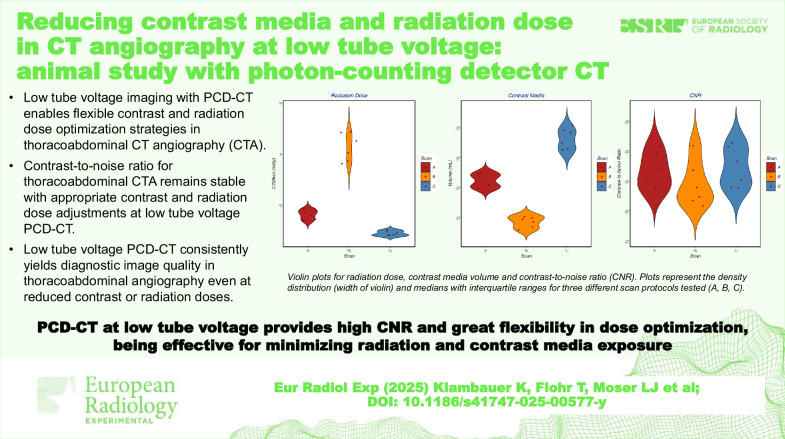

## Introduction

The continuously increasing worldwide use of computed tomography (CT) raises repeated concerns about the associated potential risks from ionizing radiation exposure and iodinated contrast media (CM) administration, which depend on CT scanners, indications, scan protocols, and patient demographics. For younger individuals or those with normal renal function, radiation exposure is a major concern [1]. However, in elderly patients or those with kidney disease, the primary concern shifts to the potential risk of CM-induced nephropathy [2].

In CT angiography (CTA), image quality (IQ) is predominantly determined by the iodine contrast-to-noise ratio (CNR), which is influenced by both CM and radiation dose. Low tube voltage CTA enhances iodine attenuation compared to standard 120 kVp protocols, allowing for reductions of either or both radiation and CM dose while maintaining the CNR. Yet, this approach requires precise optimization of scan parameters [3].

Energy-integrating detector (EID)-CT systems equipped with automated x-ray tube voltage selection (ATVS) adjust both tube voltage and tube current to the patient’s attenuation characteristics and the planned examination type, with the aim to minimize radiation dose while maintaining adequate CNR [4]. In CTA, ATVS maximizes iodine CNR for a given CM protocol by selecting the lowest possible tube voltage for the patient and the planned examination [5]. ATVS settings can be further customized to prioritize either radiation or CM dose reduction depending on clinical requirements [4]. Haubold et al demonstrated with an EID-CT scanner the feasibility of customizing CTA protocols to optimize both radiation and CM doses by using ATVS in a study involving six minipigs. Operating the CT scanner at 90 kVp with ATVS settings tailored to maximize either radiation dose or CM dose reduction, authors achieved radiation dose reductions of 30% and CM dose reductions of 26%, without significantly compromising CNR or subjective IQ [5].

Photon-counting detector (PCD)-CT represents an advanced imaging technique that enables substantial reductions in both radiation and CM doses compared to EID-CT, owing to its inherent spectral capabilities, higher contribution of low energy x-rays to the detector signal, and lower electronic noise [6]. Additionally, PCD-CT leverages low-kiloelectronvolt (keV) virtual monoenergetic images (VMI) to increase iodine CNR without necessitating a reduction in tube voltage from the standard 120 kVp/140 kVp setting for routine scanning [7, 8]. Furthermore, PCD-CT offers scanning at tube voltages of 70 kVp and 90 kVp for even more pronounced dose reductions [9].

Interestingly, no study so far has evaluated the potential of PCD-CT angiography with low tube voltage in regard to potential radiation and CM reduction, hereby expanding the previous work of Haubold et al with EID-CT [5] to PCD-CT. Thus, the purpose of this study was to investigate optimized radiation and CM doses of PCD-CT angiography at low tube voltage in an animal model.

## Materials and methods

### Animals

This experiment involved six Göttingen minipigs (Ellegaard, Dalmose, Denmark), weighing 32.5 kg (IQR: 29.8–34.6 kg). The animals were treated in accordance with German Animal Welfare Legislation, with approval from the State Animal Welfare Committee.

CT scans were conducted under general anesthesia, initiated with an intramuscular injection of 15 mg/kg ketamine (Pharmacia, Karlsruhe, Germany), 2 mg/kg azaperone (Stresnil, Elanco GmbH, Bad Homburg, Germany), and 0.02 mg/kg atropine (Eifelfango Chem.-Pharm. Werke, Bad Neuenahr-Ahrweiler, Germany). This was followed by an intravenous dose of 7 mg/kg propofol (Propofol-Lipuro, Braun, Melsungen, Germany). The animals were intubated and ventilated with an air/oxygen mixture, and anesthesia was maintained with 9–12 mg/kg/h propofol.

CTA was performed with the animals in a prone position during an end-expiratory ventilation hold, while heart rate and oxygen saturation were monitored.

### Image acquisition

Scans were performed using a first-generation, dual-source PCD-CT system (NAEOTOM Alpha, Siemens Healthineers AG, Forchheim, Germany, version VB10A) equipped with two cadmium telluride PCDs. The system was operated in single-source mode with a collimation of 144 × 0.4 mm, with a 0.25 s gantry rotation time and a tube voltage of 70 kVp. The PCD-CT provides task-based automatic keV selection and radiation dose adjustment (CARE keV, Siemens Healthineers AG, Forchheim, Germany). This mechanism follows different principles depending on the selected scan mode: For “QuantumPlus” modes with 120 kVp or 140 kVp tube voltage, the user selects the imaging task (“vascular”, “parenchyma with contrast”, “bone” or “non-contrast”) and an IQ level, and CARE keV automatically adjusts the radiation dose to achieve a constant CNR in VMIs at 55 keV, 60 keV, 65 keV, or 70 keV [10]. For “Quantum” modes with 70 kVp or 90 kVp tube voltage, the reconstruction of VMIs at different keVs is limited because of reduced spectral information. When switching from “vascular” to “non-contrast” CARE keV optimizes the fat-water CNR, instead of the iodine-water CNR, and increases the radiation dose accordingly [10]. In our study, images were then reconstructed as VMIs at 53 keV, independent of the selected imaging task. 53 keV is the optimum VMI energy at a tube voltage of 70 kVp. We used a Qr40 kernel, quantum iterative reconstruction (QIR) level 3, with a 300 × 300 mm² field of view, 0.8 mm slice thickness, and 0.5 mm increment. The quantitative kernel Qr40 was chosen because it does not enhance edges, preventing potential influences on quantitative measurements [11].

Radiation dose was estimated through the volume CT dose index (CTDI_vol_) and CM volume (mL) was recorded for each scan.

### Theoretical considerations to estimate radiation and contrast dose

The CNR is assumed to be proportional to the contrast dose *D*_C_. If CNR_A_ is obtained at contrast dose *D*_CA_, changing the contrast dose to *D*_CB_ results in

1 $${{{\mathrm{CNR}}}}_{{{{\rm{B}}}}}={D}_{{{\mathrm{CB}}}}/{D}_{{{\mathrm{CA}}}}{{{\mathrm{CNR}}}}_{{{{\rm{A}}}}}$$

The CNR is expected to be proportional to the square root of the radiation dose $\sqrt{{D}_{{{{\rm{R}}}}}}$, because the image noise σ is assumed to be proportional to $1/\sqrt{{D}_{{{{\rm{R}}}}}}$.

Changing the radiation dose from *D*_RA_ to *D*_RB_ leads to

2 $${{{\mathrm{CNR}}}}_{{{{\rm{B}}}}}=\sqrt{{D}_{{{\mathrm{RB}}}}/{D}_{{{\mathrm{RA}}}}}{{{\mathrm{CNR}}}}_{{{{\rm{A}}}}}$$

Consequently, changing the radiation dose from *D*_RA_ to *D*_RB_ must be compensated for by a change of the contrast dose to

3 $${D}_{{CB}}=\sqrt{{D}_{{RA}}/{D}_{{RB}}}{D}_{{CA}}{{{\rm{for}}}}\; {{{\rm{constant}}}}\; {{{\rm{CNR}}}}$$

### Study design

Three CTA scan protocols were evaluated in a single scan session per animal, varying the CM and radiation dose:

(A) Reference simulating standard thoracoabdominal CTA at 70 kVp. Scan at 70 kVp, IQ-level 117, with CARE keV optimized for vascular tasks. CM dose: 150 mgl/kg, flow rate: 2.5 mL/s. Mode (A) serves as the reference with radiation dose *D*_RA_ = 100% and contrast dose *D*_CA_ = 100%. The actual value of *D*_RA_ was determined as the mean CTDI_vol_ of the CT scans of the six minipigs.
(B) CTA with increased radiation dose and reduced CM dose. Scan at 70 kVp, IQ-level 117, with CARE keV optimized for non-contrast tasks. With the CARE keV functionality, the radiation dose is expected to be increased to *D*_RB_ = 2.33 *D*_RA._ The actual value of *D*_RB_ was determined as the mean CTDI_vol_ of the CT scans of the 6 minipigs. The higher radiation dose can be leveraged to reduce the contrast dose to ${D}_{{{\mathrm{CB}}}=}\sqrt{{D}_{{{\mathrm{RA}}}}/{D}_{{{\mathrm{RB}}}}}$ $x{D}_{{{\mathrm{CA}}}}$ = 0.65 ${D}_{{{\mathrm{CA}}}}$ while maintaining a constant CNR (see Eq. 3). In our experiments, we used a slightly over-proportional reduction of the CM dose to 84 mgI/kg, at a flow rate of 1.4 mL/s. Mode (B) is focused on CM dose reduction, with expected contrast dose *D*_CB_ = 56% and radiation dose *D*_RB_ = 233% compared to reference (A).
(C) CTA with reduced radiation dose and increased CM dose. Scan at 70 kVp, IQ-level 59, with CARE keV optimized for vascular tasks. Radiation dose is expected to be decreased to *D*_RC_ = 0.5 *D*_RA_ because of the manual reduction of the IQ level. The lower radiation dose needs to be compensated by a corresponding increase of the CM dose to ${D}_{{{\mathrm{CC}}}=}\sqrt{{D}_{{{\mathrm{RA}}}}/{D}_{{{\mathrm{RC}}}}}$ $x{D}_{{{\mathrm{CA}}}}$ = 1.41 ${D}_{{{\mathrm{CA}}}}$. In our experiments, we used 1.41 × 150 mgI/kg = 212 mgI/kg at a flow rate of 3.5 mL/s. Mode (C) is focused on radiation dose reduction, with expected radiation dose *D*_RC_ = 50% and contrast dose *D*_CC_ = 141% compared to the reference (A).

Scans were randomized with a 45-min CM washout between them. To ensure consistency and prevent artifacts from CM in the urinary collecting system, the scan ranges were predefined and kept identical within the same animal. The bladder was excluded from the scan area a priori based on the topogram, and no additional topograms were acquired to avoid unwanted dose modulation. Iopromide 300 mgI/mL (Ultravist 300, Bayer Vital GmbH, Leverkusen, Germany) was administered, followed by a 20 mL saline flush. The injection rate was adjusted to the CM dose as described above. Injections were performed using a power injector system (Medrad Centargo, Bayer AG, Leverkusen, Germany). Bolus tracking (90 kVp, cycle time 0.8 s) was performed using the descending aorta (trigger level = 100 HU, trigger delay = 3 s) to ensure precise scan timing.

### Objective and subjective image assessment

Objective and subjective image assessments were performed as previously reported [5]. Attenuation was measured in four circular regions of interest (ROIs) using commercial software (syngo.via, version VB10A, Siemens) in the ascending aorta, descending aorta, abdominal aorta at the level of the celiac trunk, and at the level of the renal arteries. Additionally, attenuation and image noise were measured with circular ROIs in the deep back muscles bilaterally at the renal artery level. Image noise was measured as the standard deviation (SD) of the CT numbers within the ROI. The CNR was calculated as:

4 $${CNR}=({mean} \, {aortic}\,{attenuation} - {muscle}\, {attenuation})/{muscle}\,{SD}$$

using the attenuation averages across the aortic measurements.

Two radiologists with 7 years and 4 years of experience in cardiovascular CT ([K.K.] and [L.J.M.]) independently assessed CT scans ([K.K.] performed the assessments twice). They evaluated four aspects using a 4-point Likert scale: overall IQ, vessel contrast, noise, and visibility of the distal hepatic arteries. The scale was defined as: 1 = non-diagnostic, 2 = diagnostic, 3 = good, and 4 = excellent (for noise category: 4 = least noise, best). Overall IQ and hepatic artery visibility were evaluated using noise-adapted window widths to ensure consistency across assessments:

$${Window}\,{width}\,\left({in}\,{HU}\right) = {muscle}\, {SD}\,({in}\,{HU})\, x \,50$$

**Fig. 1:**
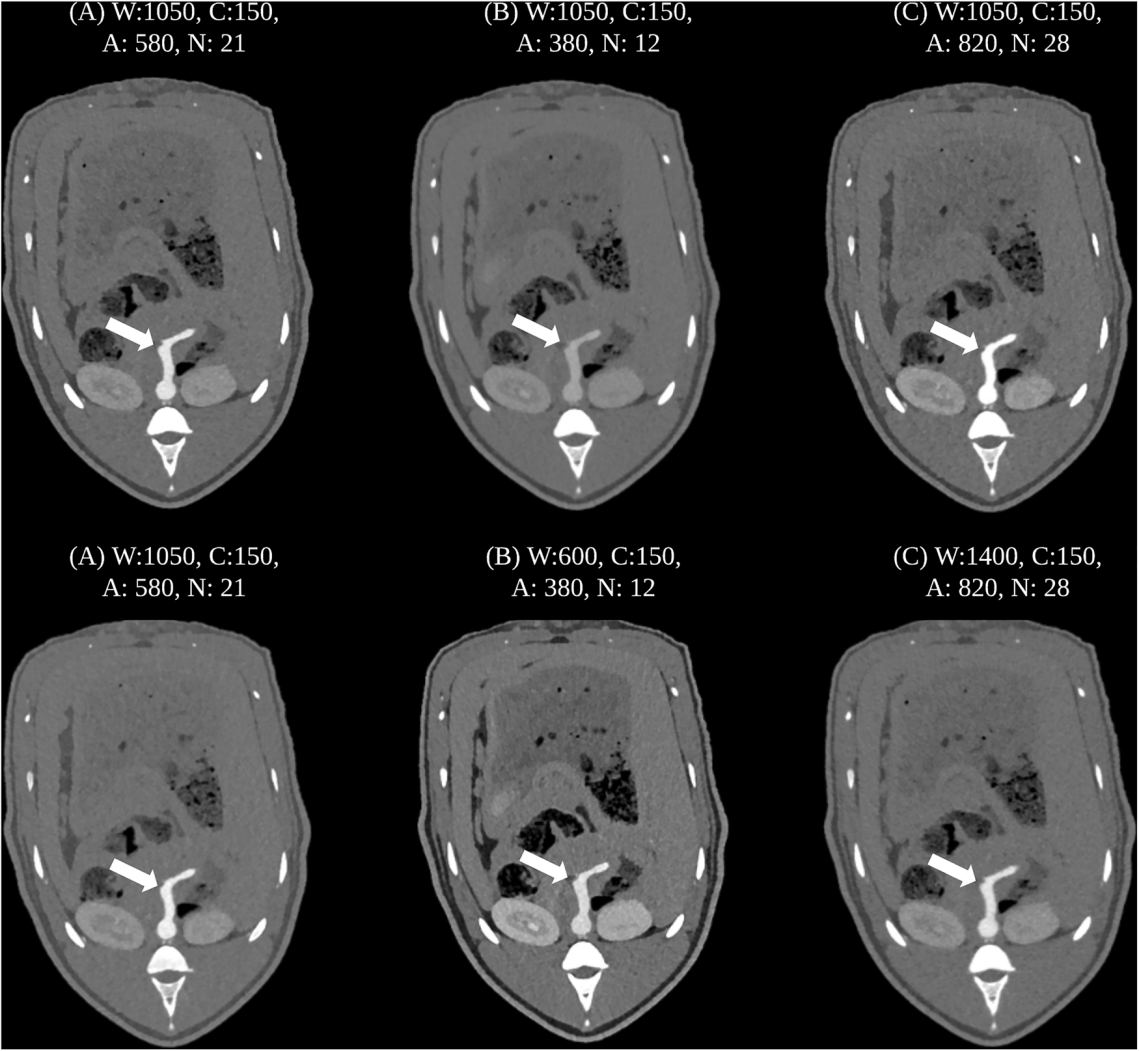
**A**–**C** Show axial images at the level of the celiac trunk of the three distinct scan protocols (**A**) scan A, (**B**) scan B, (**C**) scan C. Top row: axial images without strategic window selection. There is a difference in aortic attenuation (white arrows) when the window level is kept constant across scans. Bottom row: axial images with strategic window selection. The window level is chosen as described in the methods section, and the aortic attenuation (white arrows) appears nearly identical, hence optimizing diagnostic performance. We chose to select the window width according to the image noise with the formula noise^*^50: **A** had a noise of 21 (^*^50 = 1,050), **B** had a noise of 12 (^*^50 = 600) and **C** had a noise of 28 (^*^50 = 1,400). The window center was kept the same for all scans. A, Aortic attenuation; C, Window center; N, noise; W, Window width, all provided in Hounsfield units

This factor was determined during the initial readout process of the first animal, where a trial-and-error approach was used to identify the best compromise between image contrast and noise. Once this factor was established, it was applied uniformly across all scan readouts, and readers were not allowed to manually adjust the window level during their evaluations. This concept is visualized in Fig. 1.

### Statistical analyses

Results were reported as either median with interquartile ranges or mean with standard deviation, depending on the data distribution, which was evaluated using the Shapiro–Wilk test. Given the lack of normal distribution, the Kruskal–Wallis test was used to assess differences in scan protocols across eight parameters: attenuation in the aorta, attenuation in muscle, noise in muscle, CNR, subjective IQ, subjective vessel contrast, subjective noise, and subjective visibility of intrahepatic arteries. To account for these parameters, Bonferroni correction was applied by multiplying *p*-values by 8. For pairwise comparisons between scan protocols (Scan A *versus* B, A *versus* C, and B *versus* C) using the Mann–Whitney *U*-test, Bonferroni correction was applied by multiplying *p*-values by 3 within each parameter, avoiding overcorrection. Box, violin, and bar plots were used to display medians, interquartile ranges, and *p*-values. Cohen’s squared kappa statistic (< 0.20, no; 0.20–0.39, minimal; 0.40–0.59, weak; 0.60–0.79, moderate; 0.80–0.89 strong; and 0.89–1.00, almost perfect agreement) [12] was utilized to measure inter- and intra-reader agreement in subjective IQ evaluations. Statistical significance was determined with a *p*-value threshold of less than 0.05 after corrections. All statistical analyses were conducted using the open-source R software (version 4.4.0, R Core Team, Vienna, Austria).

## Results

### Image acquisition metrics

Scan B had the highest radiation dose (median CTDI_vol_: 4.3 mGy, IQR: 3.8–4.7 mGy) and tube current-time product (median: 280 mAs, IQR: 246–306 mAs), followed by Scan A (median CTDI_vol_: 1.7 mGy, IQR: 1.5–1.8 mGy) and tube current (median: 109 mAs, IQR: 96–119 mAs), while Scan C had the lowest radiation dose (median CTDI_vol_: 0.9 mGy, IQR: 0.8–1.0 mGy) and tube current-time product (median: 59 mAs, IQR: 52–65 mAs). The CM volumes were 16 mL (IQR: 15–17 mL) for Scan A, 10 mL (IQR: 8–10 mL) for Scan B, and 23 mL (IQR: 21–24 mL) for Scan C. Relative to Scan A, CM volumes were 63% for Scan B, and 144% for Scan C, compared to expected CM volumes of 56 and 141%. Radiation doses were 260% for Scan B and 56% for Scan C, compared to expected radiation doses of 233 and 50%. Measured vessel attenuation was 61% for Scan B and 135% for Scan C (*p* < 0.001 for all comparisons). Protocol parameters and metrics are presented in Table 1.

**Table 1:** PCD-CT image acquisition metrics

Parameters	Scan A	Scan B	Scan C
Mode (image task)	Vascular	Non-contrast	Vascular
IQ level	117	117	59
Contrast dose (mgL/kg)	150	84	212
Flow rate (mL/s)	2.5	1.4	3.5
Tube current-time product (mAs)	109 (IQR: 96–119)	280 (IQR: 246–306)	59 (IQR: 52–65)
CTDI_vol_ (mGy)	1.7 (IQR: 1.5–1.8)	4.3 (IQR: 3.8–4.7)	0.9 (IQR: 0.8–1.0)
CM volume (mL)	16 (IQR: 15–17)	10 (IQR: 8–10)	23 (IQR: 21–24)
CM volume (%)	100	63	144
Radiation dose (%)	100	260	56
Noise (%)	100	57	133
Attenuation (%)	100	61	135

Values are provided in the median and interquartile range (IQR) unless otherwise specified (for all: *p* < 0.001). Scan A was set as a reference for estimated radiation dose, noise, and attenuation calculations. Percentages for radiation dose were calculated with the CTDI

*CTDI* CT dose index, *DLP* Dose length product, *IQ* Image quality, *SSDE* Size-specific dose estimate

### Image assessment

In the objective image assessment, aortic attenuation was highest in scan C (median: 828 HU, IQR: 786–894), intermediate in scan A (median: 615 HU, IQR: 578–677), and lowest in scan B (median: 373 HU, IQR: 348–408) (*p* < 0.001). Pairwise comparisons showed significant differences between all scans (all, *p* = 0.016) for aortic attenuation. Muscle noise was lowest in scan B (median: 12 HU, IQR: 12–13), intermediate in scan A (median: 21 HU, IQR: 20–22), and highest in scan C (median: 28 HU, IQR: 28–29) (*p* < 0.001), with pairwise comparisons showing significant differences across all scans (all, *p* < 0.05). CNR was similar across all scans (*p* = 0.276). Median CNR_A_ was 26 (IQR: 24–28), median CNR_B_ 23 (IQR: 22–26), and median CNR_C_ 26 (IQR: 24–30).

All scans yielded at least diagnostic scores across all qualitative assessment categories. Subjective IQ was similar across scans, without significant differences (*p* = 0.342), with median values of 3 (IQR: 3–4) for scan A, 4 (IQR: 3–4) for scan B, and 3 (IQR: 3–3) for scan C. Subjective vessel contrast was highest in scan C (median: 4, IQR: 3–4), intermediate in scan A (median: 3, IQR: 3–4), and lowest in scan B (median: 2, IQR: 2–3) (*p* = 0.008), with significant differences between B and C (*p* = 0.027). Noise was rated worst in scan C (*p* = 0.008), with significant differences between scan B and C (*p* = 0.026), with scan A rated at 3 (IQR: 3–4), scan B at 4 (IQR: 3–4), and scan C at 2 (IQR: 2–3). Intrahepatic artery visibility did not differ significantly (*p* = 0.873), with scan A receiving a score of 3 (IQR: 3–4), scan B a score of 3 (IQR: 3–3), and scan C a score of 3 (IQR: 3–4).

The intrareader agreement was almost perfect (Cohen’s kappa: 0.862, *p* < 0.001), while the interreader agreement was substantial (Cohen’s kappa: 0.798, *p* < 0.001).

**Fig. 2:**
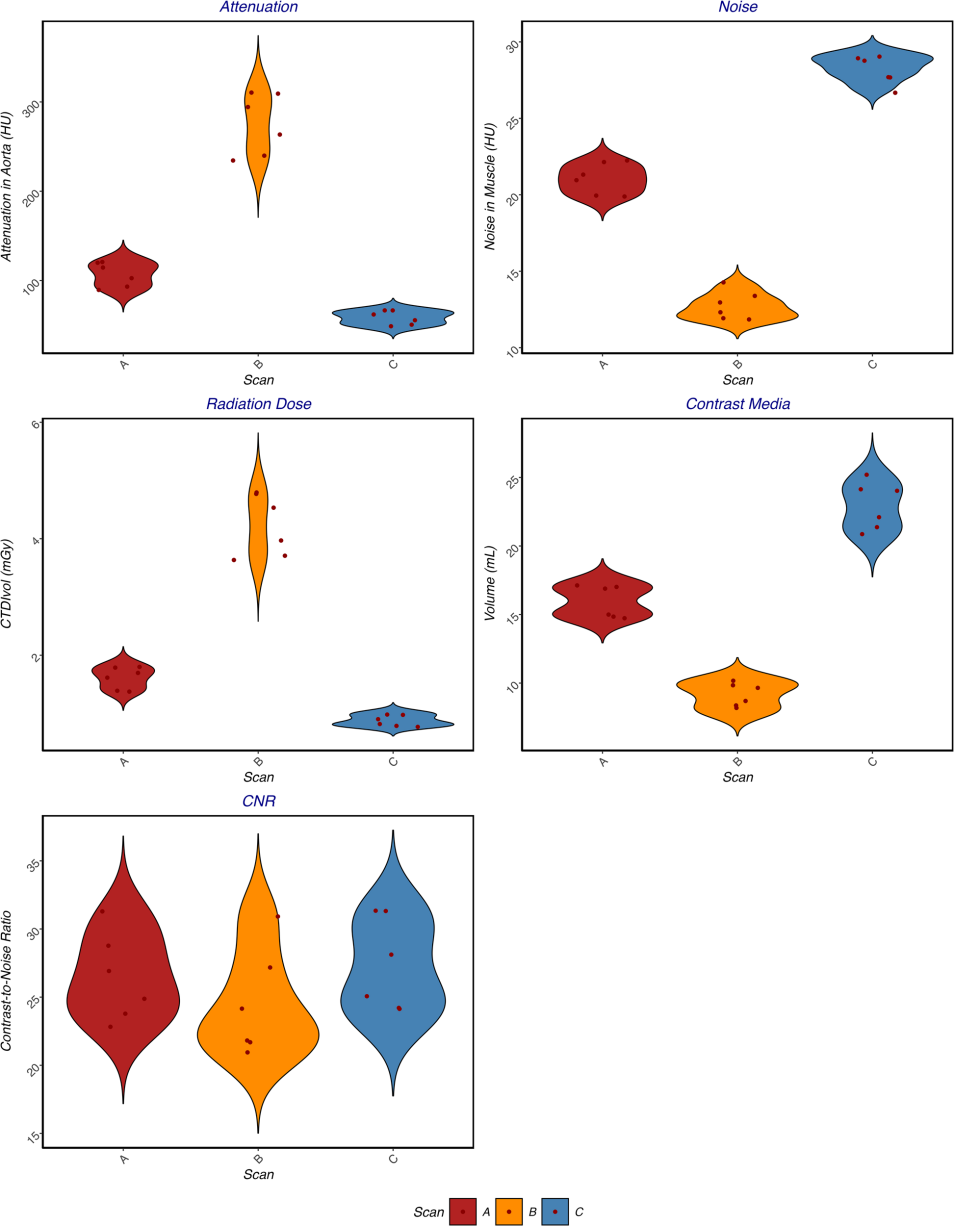
Violinplots for attenuation, noise, radiation dose, CM volume, and CNR. Plots represent the density distribution (width of violin) and medians with interquartile ranges. CTDI_vol_, Volume CT dose index

**Fig. 3:**
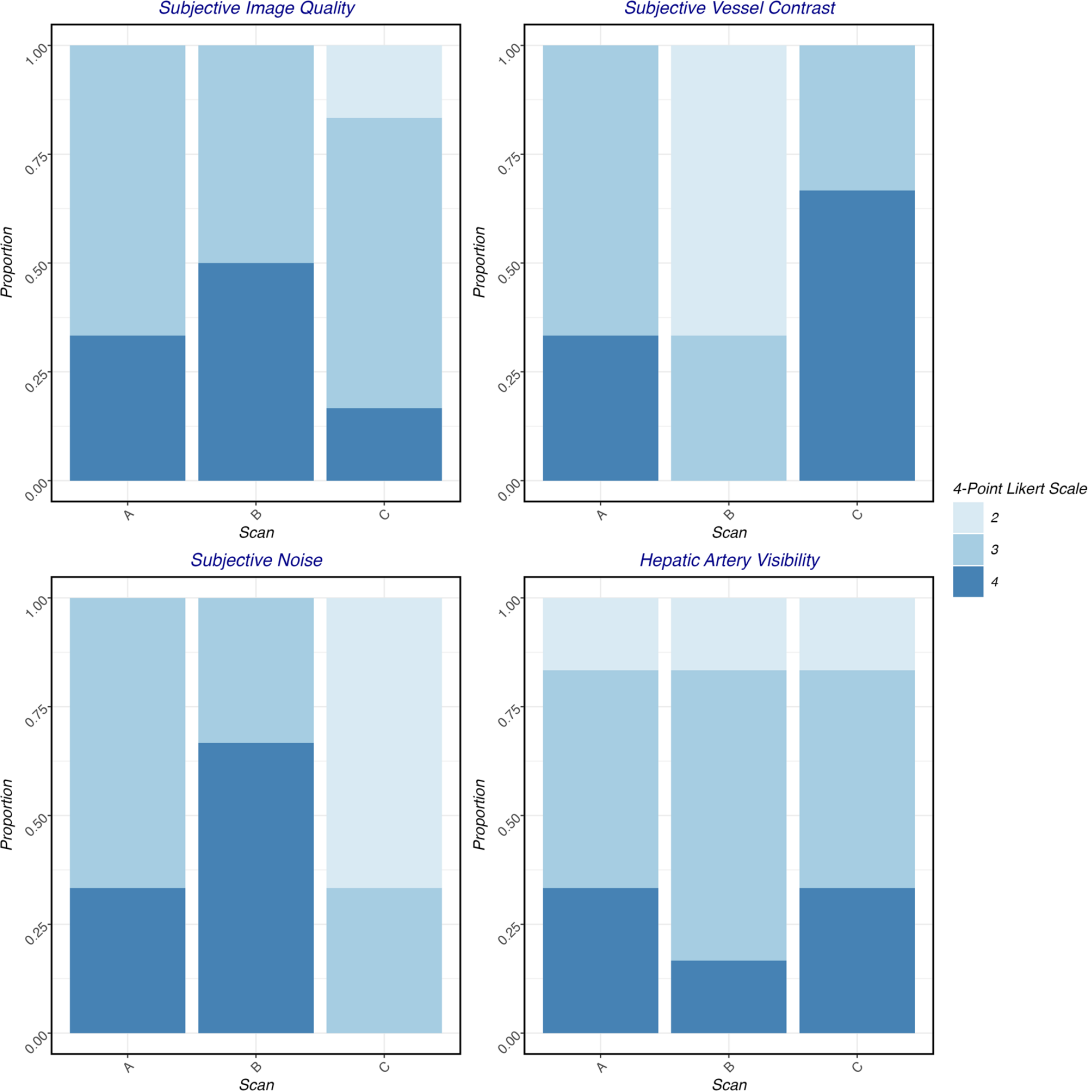
Stacked bar plots for subjective image assessment. The intrareader agreement was almost perfect (Cohen’s kappa: 0.862, *p* < 0.001), and the interreader agreement was substantial (Cohen’s kappa: 0.798, *p* < 0.001)

Objective and subjective image assessment is summarized in Tables 2 and 3 and visualized in Figs. 2 and 3.

**Table 2:** Groupwise comparison for quantitative and qualitative image assessment

Parameters	Scan A	Scan B	Scan C	*p*-values
Attenuation aorta (HU)	615 (IQR: 578–677)	373 (IQR: 348–408)	828 (IQR: 786–894)	< 0.001
Attenuation muscle (HU)	70 (IQR: 69–73)	72 (IQR: 70–72)	71 (IQR: 69–72)	0.769
Noise muscle (HU)	21 (IQR: 20–22)	12 (IQR: 12–13)	28 (IQR: 28–29)	< 0.001
CNR	26 (IQR: 24–28)	23 (IQR: 22–26)	26 (IQR: 24–30)	0.276
Subjective IQ^*^	3 (IQR: 3–4)	4 (IQR: 3–4)	3 (IQR: 3–3)	0.342
Subjective vessel contrast^*^	3 (IQR: 3–4)	2 (IQR: 2–3)	4 (IQR: 3–4)	0.008
Subjective noise^*^	3 (IQR: 3–4)	4 (IQR: 3–4)	2 (IQR: 2–3)	0.008
Subjective visibility of intrahepatic arteries^*^	3 (IQR: 3–4)	3 (IQR: 3–3)	3 (IQR: 3–4)	0.873

Values are provided in median and interquartile ranges (IQR). *p*-values after groupwise Kruskal–Wallis test

^*^ Scores range from 1 = non-diagnostic to 4 = excellent (for noise 4 = least noise, best)

*HU* Hounsfield units

**Table 3:** Pairwise comparison for quantitative and qualitative image assessment

Parameters	*p*-values Scan A *versus* B	*p*-values Scan A *versus* C	*p*-values Scan B *versus* C
Attenuation aorta (HU)	0.016	0.016	0.016
Attenuation muscle (HU)	> 0.999	> 0.999	> 0.999
Noise muscle (HU)	0.014	0.014	0.013
CNR	0.777	> 0.999	0.499
Subjective IQ^*^	> 0.999	> 0.999	0.604
Subjective vessel contrast^*^	0.055	0.936	0.027
Subjective noise^*^	0.936	0.055	0.026
Subjective visibility of intrahepatic arteries^*^	> 0.999	> 0.999	> 0.999

*p*-values after pairwise Mann–Whitney *U*-test and Bonferroni correction for multiple testing

^*^Scores range from 1 = non-diagnostic to 4 = excellent (for noise 4 = least noise, best)

*HU* Hounsfield units

## Discussion

Current PCD-CT literature mainly focuses on monoenergetic reconstructions at low kiloelectronvolt levels for optimized iodine contrast, with data acquisition performed at 120 kVp or 140 kVp. This study exploits the capability of PCD-CT to scan at low tube voltage (*i.e.*, 70 kVp) for CTA of the thoracoabdominal CTA in an animal model, with the aim to evaluate the further potential for reducing CM dose and/or radiation dose.

The key results of our study were: (i) a 44% reduction in radiation dose at increased CM volume, and a 37% reduction in CM volume at increased radiation dose, compared to the standard 70 kVp PCD-CTA scan protocol, while maintaining stable IQ with no differences in CNR across all scans; (ii) subjective assessments showed variations in contrast and noise, but overall IQ and visibility of the distal hepatic arteries as an indicator of IQ remained consistently high across scans. Adaptation of the window width to the image noise was particularly beneficial for maintaining diagnostic performance for scans with increased image noise or decreased iodine contrast, by maintaining visual CNR; and (iii) strong intra- and inter-reader agreement was achieved, affirming the reliability of IQ assessments.

Our study extended previous work with EID-CT and applied the ATVS algorithm for dose reduction in thoracoabdominal CTA on six Goettingen minipigs. Haubold et al [5] performed 90 kVp scans with following CM volumes: 27.2 mL (reference, 210 mgI/kg), 20.2 mL (CM reduction, 155 mgI/kg), and 32.2 mL (radiation reduction, 252 mgI/kg), with corresponding mean radiation doses of 2.4 mGy (reference CTDI_vol_), 4.3 mGy (CM reduction), and 1.7 mGy (radiation reduction). Images were reconstructed with sinogram-affirmed iterative reconstruction at a level of 3 and using a soft tissue convolution kernel (Bv36). Mean CNR values were 17.8 (reference), 18.2 (CM reduction), and 16.0 (radiation reduction), with a slight decline in diagnostic acceptability in the radiation-saving group, while overall IQ remained diagnostic [5].

Our study used a clinical dual-source PCD-CT system at 70 kVp in similar minipigs with lower CM volumes: 16 mL (reference), 10 mL (CM reduction), and 23 mL (radiation reduction). Our radiation doses were also lower, with median CTDI_vol_ values of 1.7 mGy for reference, 4.3 mGy for CM reduction, and as low as 0.9 mGy for radiation reduction. By doing so, we achieved higher median CNR values across all scan protocols as compared to the EID-CT study [5]: CNR of 26 as the reference, 23 for CM reduction, and 26 for radiation reduction. We used VMIs at 53 keV, which resulted in increased CNR, but we also applied a slightly sharper reconstruction kernel (Qr40), which in turn increased noise [5, 13–15]. We found that all key diagnostic parameters—CNR, overall IQ, and visibility of the distal hepatic arteries remained consistently high across all scans, without a decline in diagnostic acceptability even in the radiation reduction group.

Scan protocol C demonstrates the potential of PCD-CT to achieve substantial radiation dose reductions while maintaining diagnostic IQ through compensatory increases in CM doses. In fact, by lowering the IQ level from 117 to 59, a median CTDI_vol_ of 0.9 mGy was achieved—the lowest dose reported in this study. This approach aligns with findings by Stålhammar et al who achieved significant radiation dose reductions (CTDI_vol_ 0.32 mGy at 70 kVp) in pediatric patients with congenital heart disease, emphasizing the importance of low-kVp PCD-CT in populations requiring minimal radiation exposure such as children [9, 16–18]. Further possible applications in cardiovascular CT for scan protocol C include coronary CTA. This was recently demonstrated in a study by Araki et al, which investigated ultra-low-dose coronary CTA using PCD-CT at 70 kVp, achieving a mean CTDI_vol_ of 1.72 mGy with excellent IQ and diagnostic accuracy [19]. Notably, the capability of PCD-CT to consistently operate at 70 kVp is attributed to its reduced electronic noise and enhanced iodine contrast, enabling routine use of this tube voltage with improved IQ even at low x-ray flux [20–22]. This can be considered an advantage over EID-CT, which typically uses 90 kVp for dose reduction in CTA [10, 22, 23]. Hence, scan protocol C might be especially relevant for scenarios where prioritizing radiation dose reduction is essential and increased iodine doses are clinically acceptable, without compromising diagnostic performance.

In contrast, scan protocol B leverages an increase in radiation dose to achieve a 37% reduction in median CM volume relative to the reference. Higashigaito et al previously demonstrated that PCD-CT at 120 kVp enables a 25% CM dose reduction in abdominal CTA while maintaining non-inferior IQ compared to EID-CT at equal radiation doses [24]. Furthermore, a recent phantom study by Emrich et al showed a promising reduction of CM dose by up to 50% through appropriate VMI energy level selection alone [25]. This underscores the potential of PCD-CT to achieve even greater CM dose reductions by reducing the actual tube voltage and compensatorily increasing radiation dose. Such protocols may be particularly advantageous for patients at risk of contrast-induced nephropathy, including those with chronic kidney disease, and for oncologic patients requiring frequent imaging, where managing CM exposure is a greater concern than reducing radiation dose [2, 26–29]. Future research should focus on validating and refining low-kVp protocols tailored to these high-risk populations.

Limitations of this study include the small sample size of six pigs. While intraindividual comparison under controlled conditions can provide reliable data for quantitative measurements, the small sample size reduces the robustness of the subjective assessments. Additionally, although pigs are anatomically comparable to humans, differences in body weight and structure could limit how well the findings translate to human populations. Only 70 kVp was evaluated due to the limited number of scans permitted per minipig under animal welfare legislation; future studies may benefit from including protocols with 90 kVp. Furthermore, one experiment was conducted using a reduced CM dose of 84 mg/kg, rather than the calculated 97 mg/kg. These scans were included to evaluate whether this overproportional reduction would still maintain the CNR within acceptable limits, which was ultimately confirmed. Finally, we did not evaluate pathology, which limits the generalizability of our results to patients.

In conclusion, our study demonstrates the excellent performance of PCD-CT at a low tube voltage of 70 kVp for reducing both radiation and CM doses in CTA, hereby demonstrating the potential to tailor radiation or CM dose reduction to individual patients’ needs. With higher CNR and flexibility in dose optimization, PCD-CT at low tube voltage may be particularly effective for applications where minimizing radiation and CM exposure is more crucial than spectral imaging capabilities.

## Data Availability

The datasets used and/or analyzed during this study are available from the corresponding author upon reasonable request.

## Abbreviations

CM: Contrast media
CNR: Contrast-to-noise ratio
CT: Computed tomography
CTA: Computed tomography angiography
CTDI_vol_: Volume CT dose index
EID: Energy-integrating detector
IQ: Image quality
PCD: Photon-counting detector
VMI: Virtual monoenergetic imaging
